# Effectiveness of earplugs and noise cancelling headphones to improve turboprop acoustic comfort

**DOI:** 10.1177/10519815251333749

**Published:** 2025-04-21

**Authors:** Gerbera Vledder, Xinhe Yao, Yu (Wolf) Song, Peter Vink

**Affiliations:** 1 Delft University of Technology, Delft, The Netherlands

**Keywords:** sound, aircraft, comfort, discomfort, noise cancellation

## Abstract

**Background:**

Current jet airplanes are not sustainable, and turboprop aircraft can be a more sustainable alternative for regional travels. However, the noise levels in turboprops can range from 83 to 92 dB(A), which is higher than jets and is the largest contributor to discomfort in turboprops.

**Objective:**

The objective of this study was to assess the efficacy of utilizing noise-cancelling headphones or earplugs in mitigating (dis)comfort experienced by passengers aboard turboprop aircraft.

**Methods:**

An experiment was designed in a grounded Boeing 737 cabin with the sound source inside. Twenty-four participants experienced four conditions: jet sound (Boeing 737), turboprop (ATR 72) sound, turboprop sound with active noise-cancelling (ANC) headphones, and turboprop sound with earplugs. The sound level used for all conditions in this test ranged between 84.2 and 86.3 dB(A). Passenger experiences were measured using questionnaires, including a newly developed Ear Local Discomfort questionnaire.

**Results:**

The comfort and discomfort scores for the conditions involving ANC headphones and earplugs are significantly improved compared to the conditions without hearing protection. The impact of noise on discomfort is mitigated in these two conditions, though it remains the most prominent factor. ANC headphones cause more discomfort around the ear, while earplugs cause discomfort inside the ear.

**Conclusion:**

The use of ANC headphones and earplugs in a turboprop airplane might increase the acceptance of these airplanes. ANC headphones are slightly preferred over earplugs, but both solutions have specific limitations.

## Introduction

Turboprop airplanes can play an important role in enabling an all-electric aircraft system with almost no emissions.^1^ Even in their current configuration turboprops consume 10–60% less fuel compared to regional jet flights.^2^ However, the noise levels in turboprops are relatively high, ranging from 83 to 92 dB(A) depending on the seat location in the aircraft, flight phase, and aircraft type.^3^

Noise might reduce the total comfort and increase the discomfort of passengers. A good comfort rating is related to passengers’ willingness to fly with a certain airline.^4^ According to Bouwens,^5^ comfort in a jet engine airplane is dependent on the seat, noise, light, temperature, vibrations, and smell, in descending order of importance. Vink et al.^6^ found that noise is the largest contributor to discomfort in a turboprop aircraft.

Auditory comfort is a complex notion integrating subject-related factors (e.g., noise sensitivity, prior experience), situational factors (e.g., context, the task at hand), and physical factors.^7^ Quehl et al.^8^ connected auditory comfort to the absence of annoyance. Technically there is no difference between sound and noise, although the distinction is relevant for the human listener.^9^ “Noise is unwanted sound, or more precisely: noise is unwanted sound given one's current needs, goals, and activities.”.^10^ Therefore it is difficult to define noise by amplitude or frequency as it might be different for everyone, and is depending on the activity. Sounds with prominent tones^8^ and fluctuations in pitch are often very annoying.^11^ Additionally, the influence of low-frequency noises (10 Hz to 200 Hz) on annoyance is often underestimated.^12^

Fink^13^emphasizes the harmful effects noise can have on people and animals. Noise exposure can have a direct influence on people by inducing hearing loss, when exposed to high sound levels, and can have an indirect effect (non-auditory effects) which manifests in annoyance, and disturbance of e.g., concentration, relaxation, or sleep.^14^ Sound pressure levels (SPL) are often used to define the amplitude of the noise. Sleep is disrupted at 30 dB(A) and concentration at 45 dB(A).^13^ The threshold for physical discomfort (loudness discomfort level) is in the range of 80–100 dB SPL.^9^ Occupational safety regulations restrict the noise exposure to be maximally 85 decibels averaged over 8 working hours, measured as a time-weighted average (TWA).^15^ Above 85 dB(A) employees are obliged to wear hearing protection.^16^ Regarding the frequency, interior noise in turboprop airplanes is attributed to a dominant tonal component in the low-frequency band, in the case of an ATR 72–500 around 100 Hz^17^ which is generated by the blade passage frequency.^18^

Passive hearing protection nor active noise cancelling (ANC) headphones are new inventions, with the latter being developed by the U.S. Air Force more than 50 years ago.^19^ Hearing protection exists in the form of earmuffs or earplugs, both with options for active or passive hearing protection. Certain product families for external-ear worn products can be defined as over-the-ear headphones, around-the-ear headphones, in-the-ear headsets, behind-the-ear headsets, and in-the-ear headsets with ear hooks.^20^

Passive hearing protection reduces noise and filters frequencies by using noise-blocking materials, while active hearing protection cancels out noise by generating sound waves that precisely match and reverse the frequency and amplitude of the incoming noise.^21^ Where passive hearing protection works best in attenuating high-frequency noises (>500 Hz),^22^ ANC systems are very efficient in attenuating low-frequency noises.^23^ For instance, a motor helmet with ANC can effectively reduce motorcycle engine noise by 40 dB in the range <200 Hz, and by 15 dB between 200 to 600 Hz.^23^ Though the performance of ANC varies among different types of products,^22^ earplugs and ANC headphones need to meet the standard of a minimal noise reduction of 12, 11, and 9 dB(A) for compliance.^24,25^

While controlling noise levels with earplugs may enhance the comfort of passengers in jet engine airplanes,^26^ it also presents challenges related to comfort, convenience, cost, communication, and corporate culture and safety.^19^ For instance, prolonged use of headphones may lead to discomfort around the ears. When evaluating the comfort of earplugs, people first consider if the earplugs cause physical discomfort, followed by the functional fit and effectiveness, the satisfaction and well-being, and the acoustical attributes (e.g., intelligibility of alarm signals, and discomfort due to internal noises).^27^

The study's objective is to assess the effectiveness of various noise cancellation methods in turboprop flights in terms of passenger comfort. This inquiry leads to the research question: What is the impact of using noise-canceling headphones or earplugs on passenger comfort and discomfort in turboprop airplanes?

## Materials & methods

### Participants

Twenty-four participants with prior air travel experience were recruited, although not all of them had experience with turboprops (Experience: 37.5%, No experience: 16.7%, Uncertain: 45.8%). Participants with self-reported hearing impairments were excluded from the study. The sample size of twenty-four participants was determined based on a power analysis conducted prior to the study (power = .95, alpha = .05, effect size = .8), comparing the means of two dependent groups using non-parametric tests in a two-tailed setup. Table 1 displays the participants’ demographics. Anthropometric measurements were recorded following the procedure by Molenbroek et al.^28^ Two participants did not fill out all questions for unknown reasons.

**Table 1.** Participant details and anthropometric measurements.

Total number	24
Age	27 ± 4.016
Sex	12 Male/12 Female
Stature (mm)	1684 ± 85.675
Mass (kg)	69 ± 16.159
Hip width (mm)	369 ± 39.563
Popliteal height (mm)	474 ± 34.926
Buttock popliteal depth (mm)	499 ± 37.329

### Materials

**Figure 1.**
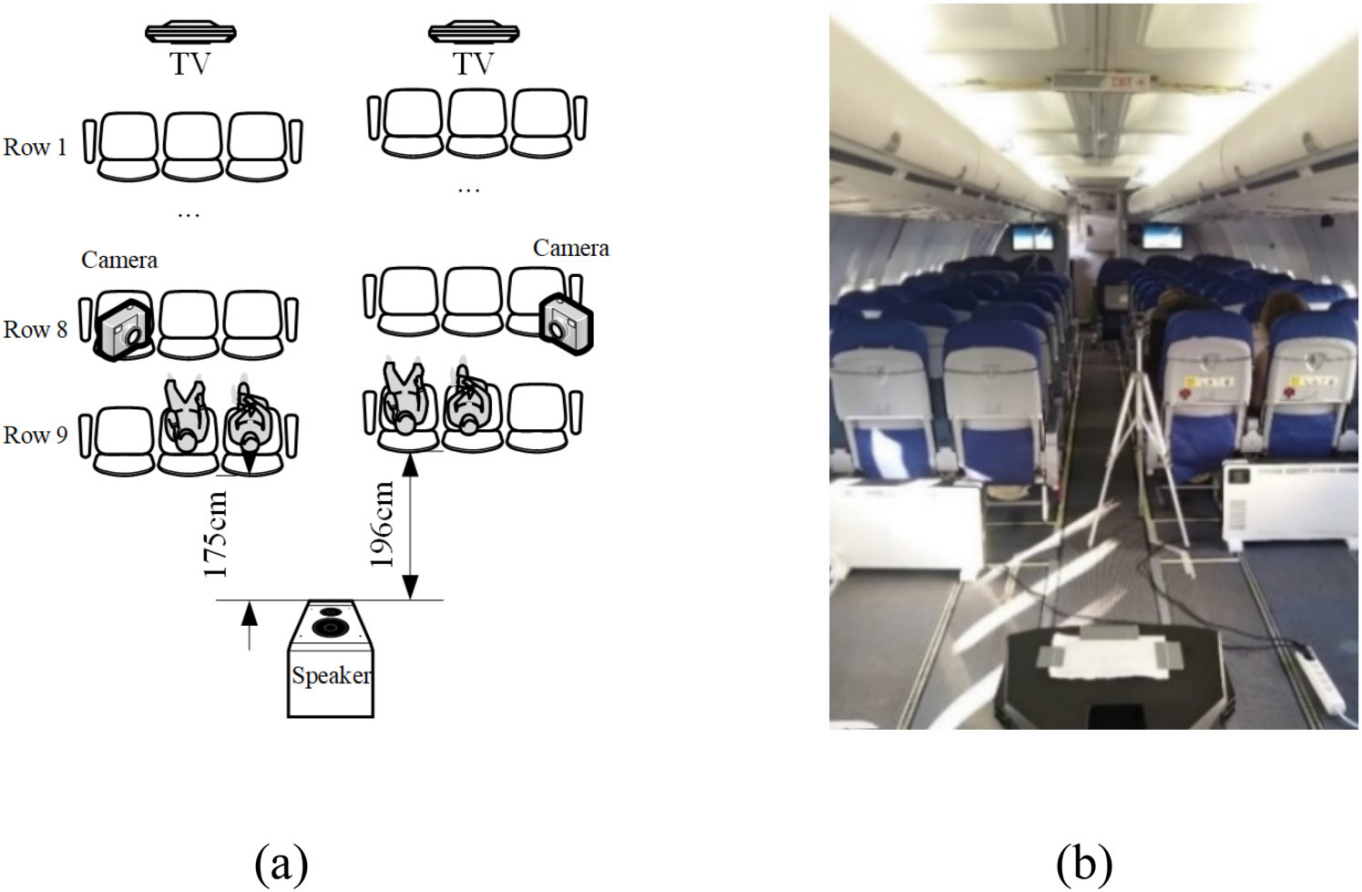
Experiment setup.

Figure 1(a) depicts the experimental setup within a grounded Boeing 737 cabin. A maximum of four participants joined the experiments simultaneously, seated in 9B, 9C, 9D, and 9E. A loudspeaker (Type: Mackie Thump 15BST) was positioned behind Row 9. The distance between the speaker and Row 9, as well as the volume of the speaker, were controlled to replicate the conditions recorded in a real ATR 72–500 flight:^17^ 82.6 dB(A) at the Row 9 aisle seats (Seats 9C and 9D), and 84.6 dB(A) for the window seats (Seats 9B and 9E). Figure 1(b) shows a photo of the experiment setup.

**Figure 2.**
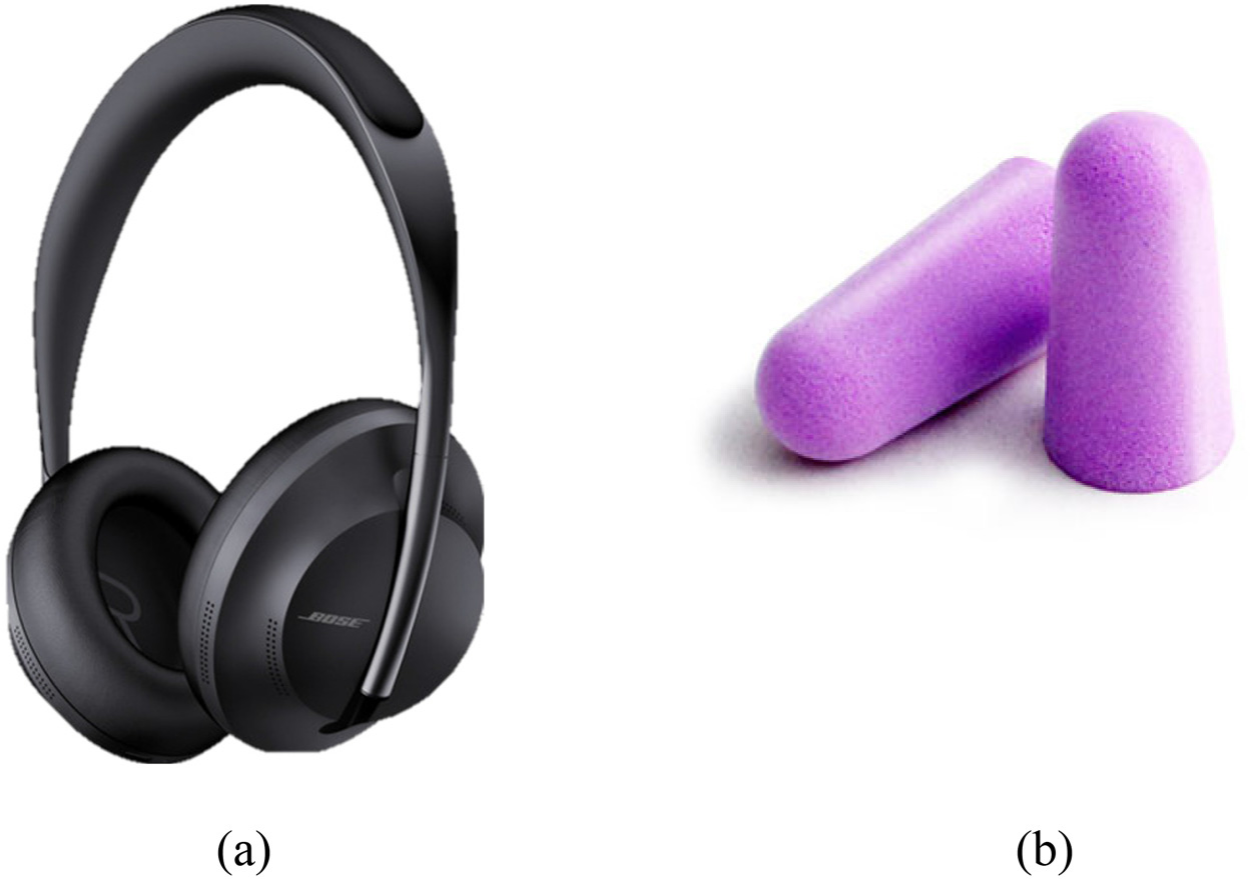
Bose^®^ noise cancelling 700 headphones (a), and Mack's^®^ slim fit soft foam earplugs (b).

Four distinct conditions were established, with the average SPL distribution (measured in dB(A)) across the seats presented in Table 2. Condition 1 (C1) served as the control scenario, featuring a jet sound recording (Boeing 737) played at the same SPL as the turboprop. In Condition 2 (C2), a turboprop sound recording (ATR 72–500) was used. Conditions 3 (C3) and 4 (C4) introduced turboprop sounds, along with ANC headphones and earplugs, respectively. In Condition 3 and 4, a Bose^®^ NC 700 (Figure 2(a)) and Mack's^®^ Slim Fit Soft Foam earplugs (Figure 2(b)) were used, respectively. The choice for the Bose^®^ NC 700 headphones was based on an evaluation that it was selected as the most comfortable headphones on the market at that time.^29^ The Mack's^®^ Slim Fit Soft Foam earplugs were selected since this earplug is the most comfortable and best for filtering out airplane noise according to the New York Times.^30^ The seats used were the current KLM Boeing 737s Recaro seats.

**Table 2.** Average SPL of each seat. Unit is in dB(A), measured by B&K^®^ 2270 Sound Level Meter.

Seat Number	9B	9C	9D	9E
Jet engine recording (Boeing 737 sound, with turboprop SPL)(C1)	84.6	86.1	86.0	84.2
Recording Turboprop (ATR 72-500)(C2, 3 and 4)	86.3	84.9	84.8	86.3

### Questionnaires

**Figure 3.**
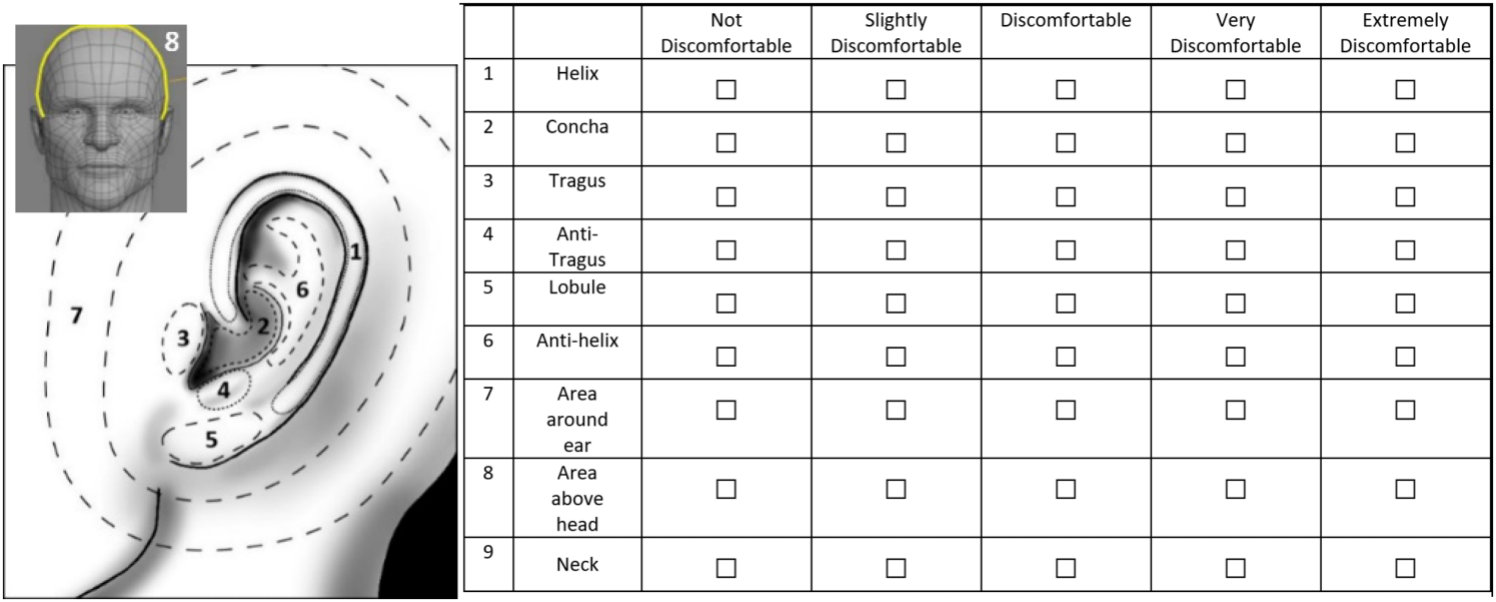
Human ear/head areas as used in Q4 (ELD), based on Stavrakos et al.^20^ and LPD.^31^

Seven types of questions/questionnaires were employed in the experiment, and they are listed in Table 3. In addition to these existing questionnaires, a newly developed Ear Local Discomfort (ELD) questionnaire (Q4) was used to assist participants in localizing discomfort around the ear, as shown in Figure 3. The regions around the ear were defined based on ear anatomy and critical interaction areas for over-ear headphones and in-ear headsets, following the descriptions of Stavrakos et al..^20^ The Likert scale used in the ELD questionnaire was inspired by the Local Postural Discomfort (LPD) questionnaire.^31^ All questions were asked while the participants were still in the airplane in the seat to prevent that memory will play a role in scoring as Mansfield et al.^32^ showed that memory errors can creep in once a participant leaves their seat.

**Table 3.** Questions/questionnaires asked during the test.

Question number	Question	Rating scale	Reference
Q1	Rank the different conditions according to your preference.		Self-composed
Q2/Q3	Give your overall comfort/discomfort rating over the last 42 min.	Scale of one to ten. 1 = no comfort, 10 = extreme comfort	Adopted from Anjani et al.^31^
Q4	Ear Local Discomfort (ELD) using the image in Figure 3. please identify your discomfort for each body area indicated.	1 = not discomfortable, 2 = slightly discomfortable, 3 = discomfortable, 4 = very discomfortable, 5 = extremely discomfortable.	Based on the local posture discomfort questionnaire from Anjani et al.^31^
Q5	Identify anything which is causing you discomfort at this moment.	Open question	Adopted from^6^
Q6	Mark the three factors most contributing to your experienced level of discomfort.	Choose from the options: Temperature, Noise, Lighting, Air quality, Vibration, Seat, Space	Adopted from,^6^ based on^5^
Q7	Why would or wouldn’t you want to wear ANC headphones or earplugs again on an airplane?	Open question	Self-composed

### Protocols

**Figure 4.**
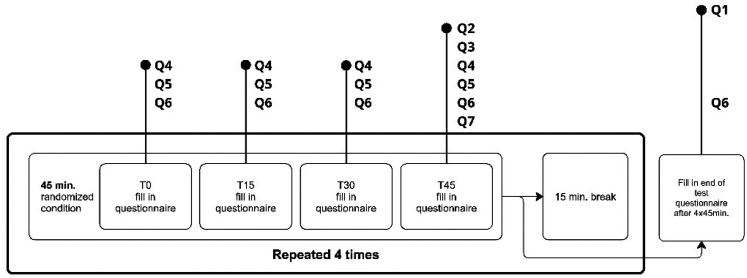
Research protocol.

The research protocol is shown in Figure 4. After acquiring informed consent, the basic anthropometrics of each participant were measured according to the procedure described by Molenbroek et al.^28^ Participants underwent four sessions, each taking place in the same seat. Each session consisted of 45 min of noise exposure under one of the four conditions using the Latin square to define the order, followed by a 15-min break. At specific time intervals within each session (T0, T15, T30, and T45), participants were required to complete questionnaires as outlined in Figure 4. Additionally, they were asked to fill out Q1 and Q6 at the end of all sessions. Besides filling out questionnaires, participants had the option to relax or engage in activities like reading from a tablet, phone, or book. After each condition, participants were instructed to leave the fuselage temporarily, take a short walk, visit the restroom, or have a drink. They were not allowed to remain in their seats during this time. To ensure the correct usage of the earplugs, participants were provided with instructions.

### Data analysis

The data analysis was conducted using SPSS^®^ 28. The normality of the data was assessed using the Shapiro-Wilk test. Differences between normally distributed data were analyzed using the paired t-test, while the Wilcoxon signed-rank test was employed for non-normally distributed data. For questions Q1, Q2, Q3, and the ELD score over time a Bonferroni correction was added. Additionally, correlations for the ELD scores, the comfort/discomfort scores, and participant anthropometrics in C3 and C4 were determined using the Pearson correlation coefficient.

## Results

**Figure 5.**
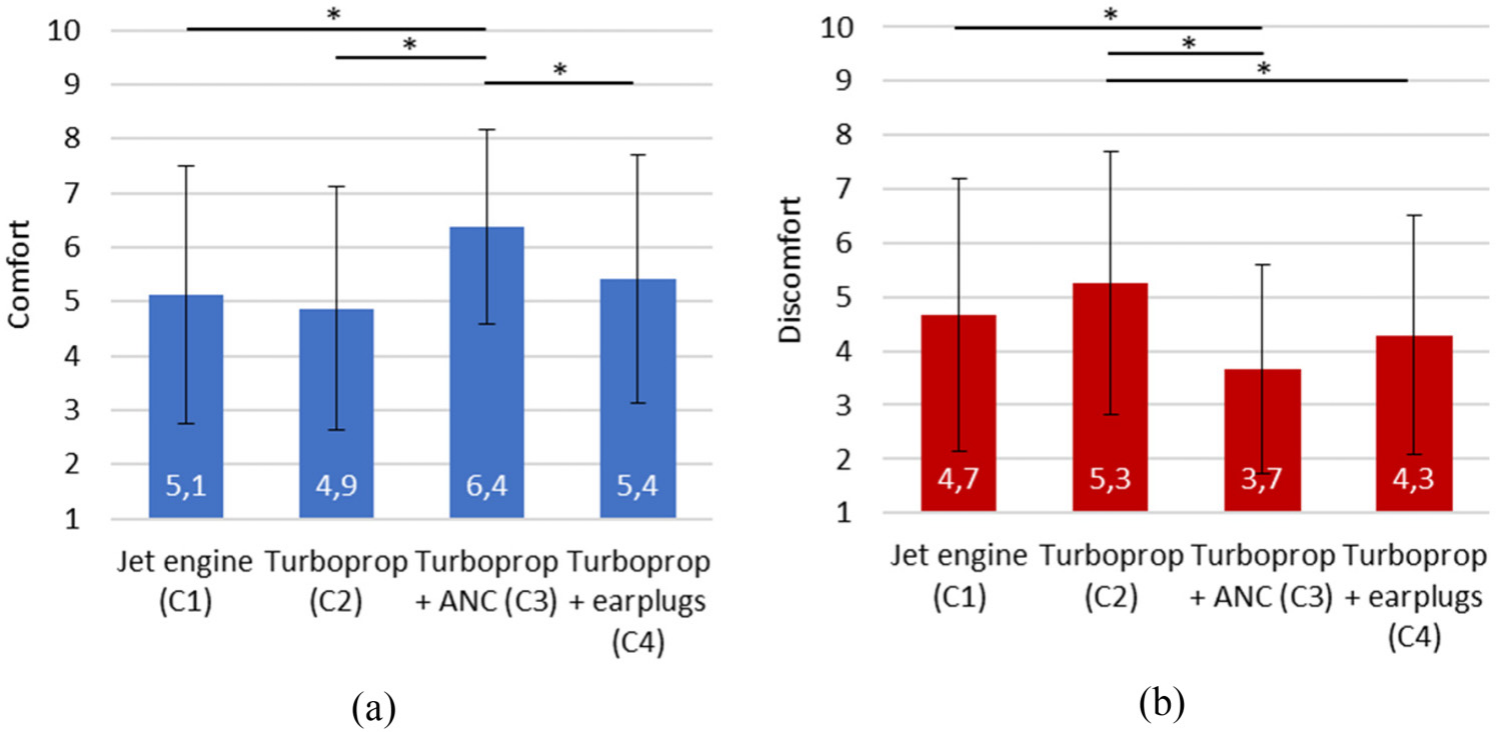
Comfort (a) and discomfort (b) rating per condition after 45 min., 1 = no comfort, 7 = extreme comfort, 1 = no discomfort, 7 = extreme discomfort. Significant (p < .05) results are marked with *.

**Figure 6.**
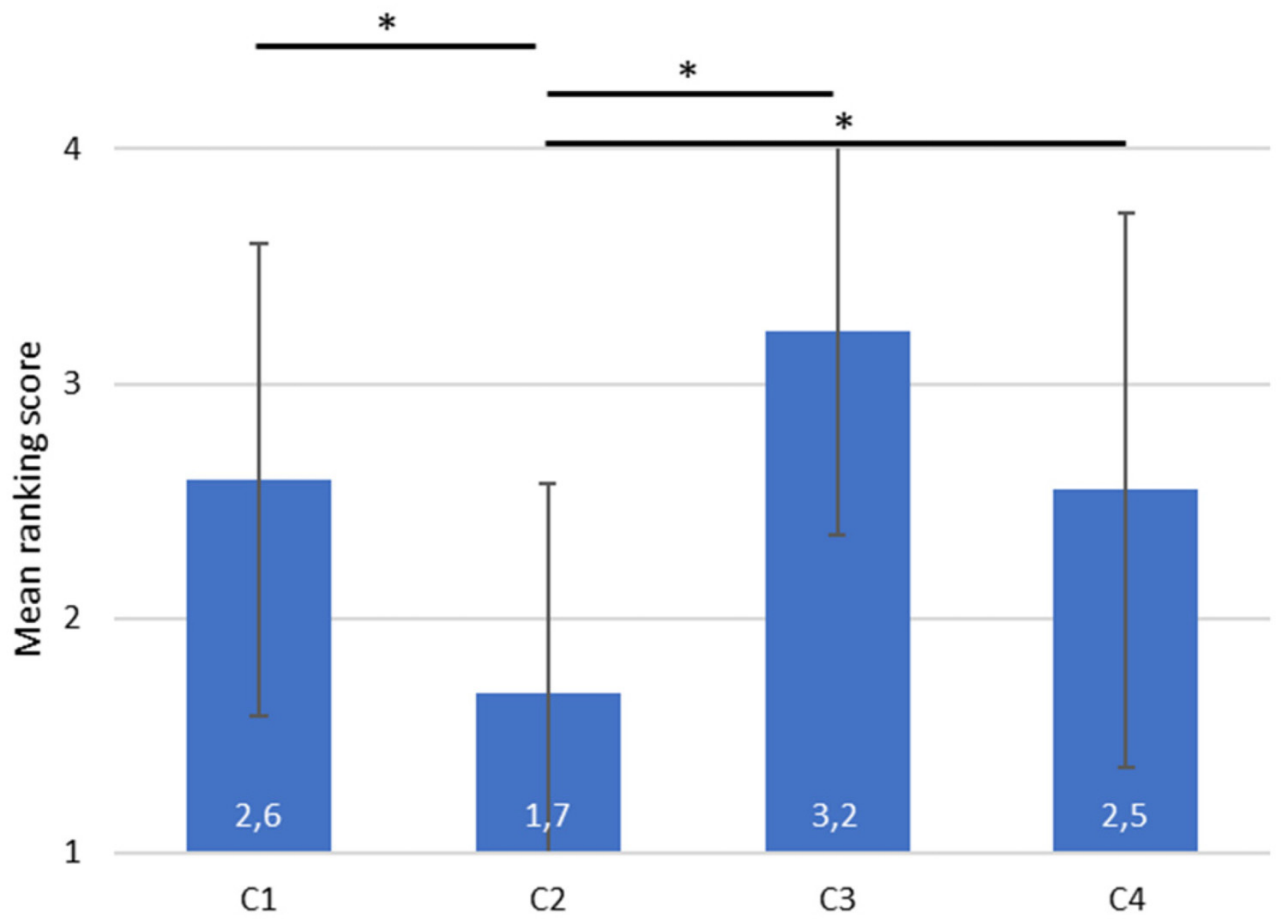
The condition preference at the end of the test (Q1). The ranked conditions were given a score where ranking 1 would get 4 points, 2 = 3, 3 = 2 and 4 = 1. Significant (p < .05) results are marked with *.

The outcomes of the Wilcoxon signed rank and paired t-tests for the comfort, discomfort, and preference scores are presented in Table 4. Table 4 shows the p-value with and without the Bonferroni correction, for the following data description the p-values without the correction are used. Participants reported statistically significant (p < .05) higher comfort, and lower discomfort scores for C3 and C4 compared to C2, as shown in Figure 5. This trend is supported by the preference score (Q1) at the end of the test (Figure 6).

**Table 4.** Q1, Q2 and Q3 pairwise comparison of conditions. P-values for the paired t-test of the comfort score. P-values for the Wilcoxon signed ranks test of the discomfort and the preference score. Values with a Bonferroni correction are added on the right side.

	Comfort (N = 24) Sig. (2-tailed)	Discomfort (N = 24) Sig. (2-tailed)	Preference (N = 22) Sig. (2-tailed)
Turboprop (C2) – Turboprop + ANC (C3)	<.001*/<.001*	<.001*/.005*	<.001*/.004*
Turboprop (C2) – Turboprop + Earplug (C4)	.102/.614	.032*/.194	.025*/.151
Turboprop (C2) – Jet Engine (C1)	.472/1.000	.165/.991	.009*/.051*
Turboprop + ANC (C3) – Turboprop + Earplug (C4)	.017*/.104	.127/.764	.058/.349
Turboprop + ANC (C3) – Jet Engine (C1)	.001*/.008*	.017*/.105	.059/.352
Turboprop + Earplug (C4) – Jet Engine (C1)	.484/1.000	.505/1.000	.908/1.000

*Significant: p < .05.

Furthermore, the condition involving ANC headphones (C3) demonstrated higher comfort and lower discomfort than the jet engine sound (C1), with a significant difference (p < .05). However, when it comes to preference, the difference is not statistically significant. In terms of comfort, ANC headphones outperform earplugs (p < .05). For discomfort and preference, similar trends were observed, although the differences are not statistically significant.

**Figure 7.**
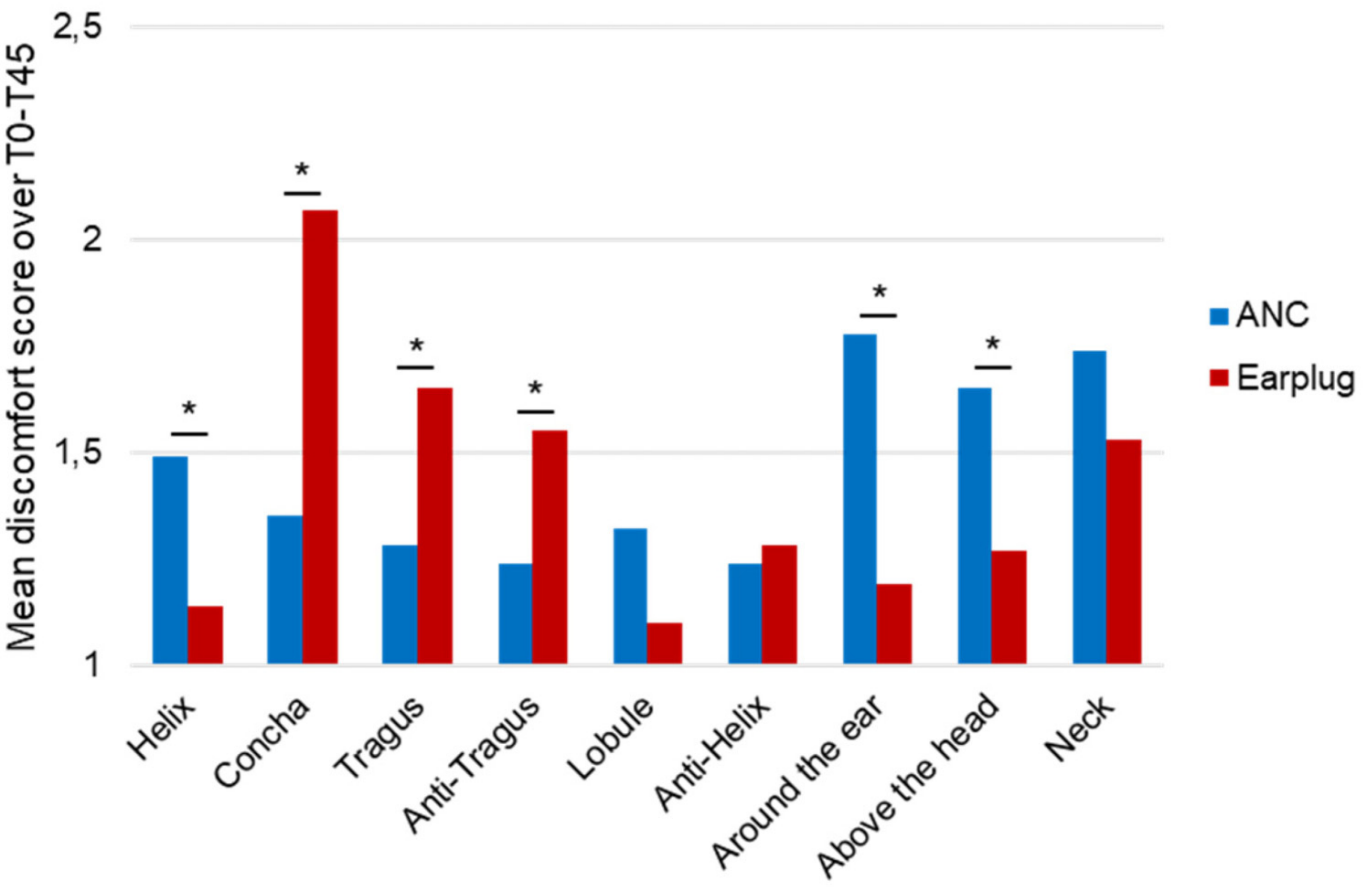
Ear local discomfort (Q4) mean over time, significant (p < .05) results are marked with *.

**Figure 8.**
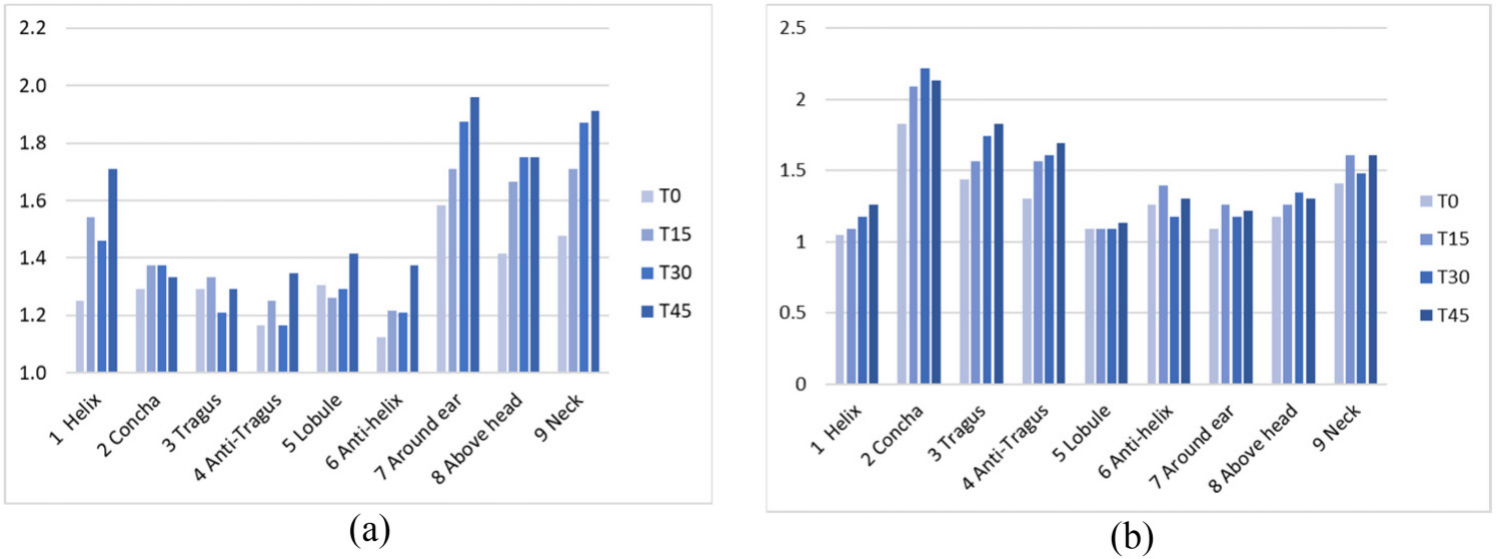
Ear local discomfort (Q4) over time of C3 ANC headphones (a) and C4 earplugs (b).

The ELD scores reveal that while the overall discomfort scores are relatively low (just above and under 2 on a 5-point scale), there are significant differences in various regions between using headphones and earplugs, as shown in Figure 7. When using earplugs, participants reported significantly higher scores (p < .05) in the inner ear regions such as Concha, Tragus, Anti-Tragus, and Anti-Helix than when using ANC headphones. On the other hand, using ANC headphones resulted in significantly higher discomfort scores (p < .05) in the areas around the ear, e.g., Helix, Lobule, Around the ear, Above the head, and Neck (Figure 7). Over time, there are significant increases (based on uncorrected results) of discomfort for the helix, the anti-helix, around the ear, above the head, and neck area while wearing ANC headphones and for the concha, tragus, anti-tragus, and around the ear while wearing earplugs, as shown in Table 5. A comparison of the different discomfort scores over time is also visualized in Figure 8.

**Table 5.** Uncorrected p-values Wilcoxon signed rank test, testing changes over time in ELD scores.

	ANC						Earplugs					
(Sig.2 tailed)	T0-15	T0-30	T0-45	T15-30	T15-45	T30-45	T0-15	T0-30	T0-45	T15-30	T15-45	T30-45
Helix	.084	.190	.026*	.317	.157	.034	.317	.180	.102	.317	.102	.157
Concha	.414	.480	.739	1.000	.655	.705	.034*	.029*	.124	.180	.763	.480
Tragus	.705	.317	1.000	.257	.705	.157	.257	.052	.029*	.046*	.034*	.317
Anti-Tragus	.414	1.000	.157	.414	.083	.102	.058	.035*	.021*	.655	.257	.157
Lobule	.317	.705	.763	.564	.257	.083	1.000	1.000	.564	1.000	.564	.317
Anti-Helix	.414	.414	.096	1.000	.083	.046*	.414	.317	.739	.157	.726	.180
Around the ear	.257	.071	.038*	.102	.083	.480	.046*	.317	.257	.157	.564	.317
Above the head	.034*	.011*	.033	.317	.317	1.000	.157	.157	.083	.414	.317	.785
Neck	.014*	.014*	.004**	.317	.102	.655	.206	.739	.160	.083	1.000	.334

*Significant: p < .05.

**Significant after Bonferroni correction.

Tables 6 and 7 display the Pearson correlation coefficients among the ELD scores, the comfort/discomfort scores, and participant anthropometrics in C3 and C4, respectively. In the scenarios using ANC (C3), we observed weak (>0.3) to moderate correlations (>0.5) between the ELD scores and the overall discomfort scores, along with inversely weak correlations (<−0.3) to moderate correlations (<−0.5) between the ELD scores and the overall comfort scores. However, in the case of earplugs (C4), such relationships were not observed.

**Table 6.** Pearson correlation coefficient of turboprop with ANC headphones Q2, Q3, and Q4.

ANC Headphones	Helix	Concha	Tragus	Anti-Tragus	Lobule	Anti-Helix	Around the ear	Above the head	neck	Overall Comfort	Overall Discomfort
Concha	0.724										
Tragus	0.677	0.569									
Anti-Tragus	0.44	0.57	0.724								
Lobule	0.511	0.419	0.648	0.478							
Anti-Helix	0.5	0.606	0.823	0.807	0.505						
Around the ear	0.657	0.617	0.536	0.31	0.487	0.403					
Above the head	0.583	0.461	0.717	0.476	0.487	0.614	0.704				
neck	0.682	0.761	0.599	0.693	0.238	0.601	0.639	0.667			
Overall Comfort	−0.21	−0.371	−0.327	−0.438	−0.204	−0.502	−0.411	−0.485	−0.548		
Overall Discomfort	0.237	0.386	0.372	0.454	0.216	0.537	0.39	0.434	0.536	−0.934	
Stature	−0.196	−0.236	−0.01	−0.029	0.033	−0.002	−0.156	−0.1	−0.168	0.1	−0.089
Age	−0.246	−0.119	−0.105	0.045	−0.247	0.084	−0.156	0.048	0.012	−0.24	0.082

**Table 7.** Pearson correlation coefficient of turboprop with earplugs headphones Q2, Q3, and Q4.

	Helix	Concha	Tragus	Anti-Tragus	Lobule	Anti-Helix	Around the ear	Above the head	neck	Overall Comfort	Overall Discomfort
Concha	0.588										
Tragus	0.449	0.669									
Anti-Tragus	0.558	0.67	0.911								
Lobule	0.937	0.556	0.397	0.591							
Anti-Helix	0.513	0.503	0.71	0.816	0.62						
Around the ear	0.692	0.594	0.217	0.408	0.769	0.359					
Above the head	0.704	0.533	0.618	0.776	0.775	0.831	0.647				
neck	0.424	0.579	0.425	0.519	0.583	0.644	0.758	0.66			
Overall Comfort	−0.077	−0.007	−0.098	−0.175	−0.144	−0.093	−0.057	−0.284	−0.027		
Overall Discomfort	−0.023	−0.059	0.077	0.16	0.054	0.095	−0.075	0.229	−0.037	−0.867	
Stature	−0.225	−0.464	−0.069	−0.091	−0.205	−0.151	−0.394	−0.327	−0.292	0.231	−0.141
Age	−0.066	−0.297	−0.168	−0.201	0.007	0.016	−0.042	0.055	0.041	−0.101	0.029

Table 8 gives a qualitative overview of Q5 and Q7. Most participants mentioned that the disadvantages of using ANC headphones are pressure on the head, weight, neck pain, vibration, sweating, and a feeling of low air pressure. For earplugs, besides blocking social communications (reduced speech recognition), participants reported itchiness and an unfamiliar feeling around the Concha region.

**Table 8.** Advantages and disadvantages deducted from Q5 and Q7.

	ANC headphones	Earplugs
Advantages	- Enables listening to entertainment - Effective noise cancellation - Clean - Easy to remove - Helps to focus on a task (e.g., reading)	- Lightweight - Easy to wear - Does not add pressure on the head
Disadvantages	- Puts pressure on the head and around the ears - Weight of the headphones - Neck pain due to the weight - Vibration is noticed more and can lead to an uncomfortable feeling to the heart. - Bad speech recognition - Sweating on the anti-helix - Feeling of low air pressure around the ears - Breaking of the ‘sound’ seal after jaw movements	- Itchy - Bad speech recognition - Unfamiliar feeling in the Concha

**Figure 9.**
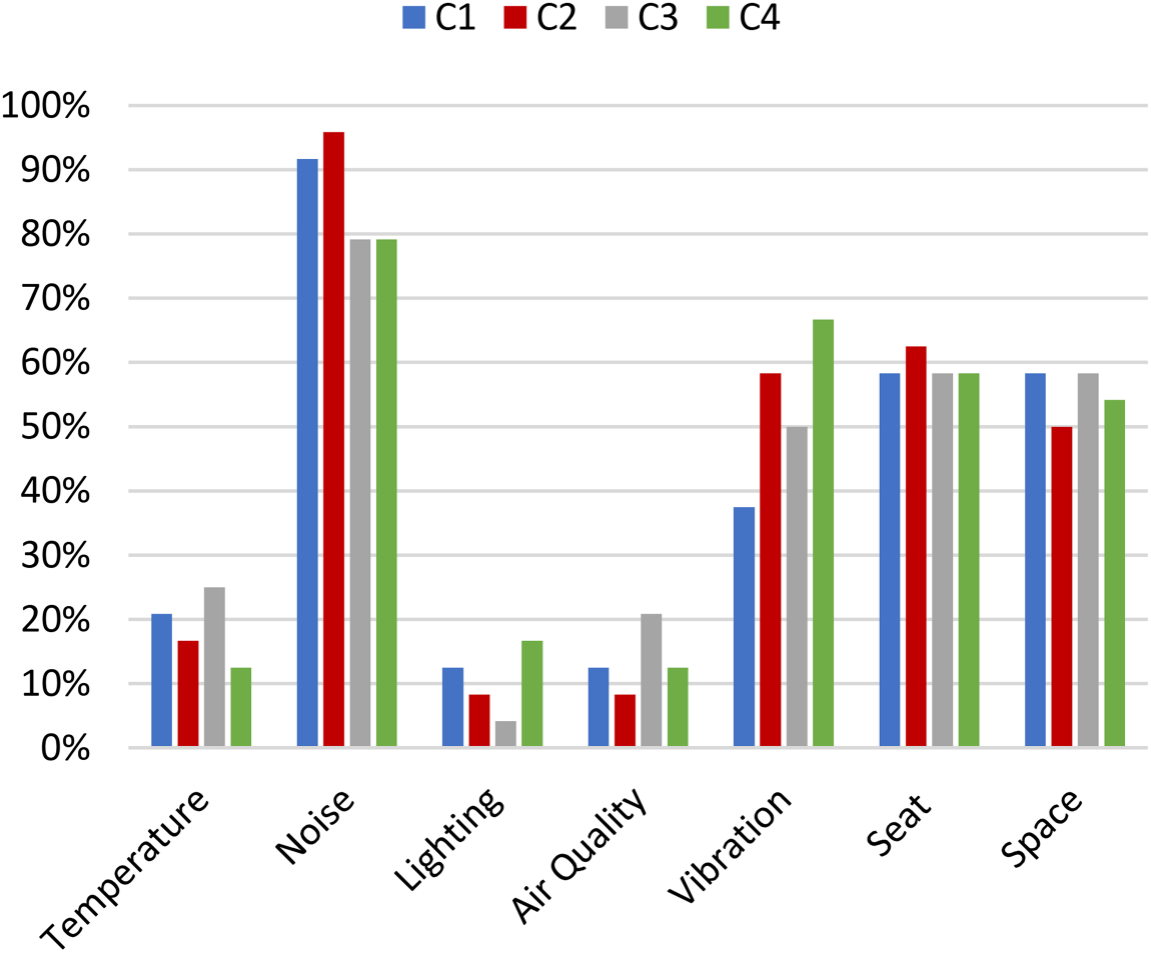
Percentage of 24 participants choosing the factors contributing to the experienced level of discomfort at T45 (Q6).

In Figure 9, the opinions of the participants regarding the factors that contribute to discomfort at T45 are shown (Q6). While wearing ANC headphones and earplugs, the impact of noise on the overall discomfort is reduced, but it remains the most important factor that influences discomfort. We did not find differences between using ANC headphones and earplugs.

## Discussion

The use of either ANC headphones or earplugs has a positive effect on passenger comfort and discomfort by enhancing comfort and reducing discomfort. While there is a preference for ANC headphones over earplugs, it is important to note that this difference is not statistically significant. The ELD scores help define specific discomfort areas for both earplugs and ANC headphones. The discomfort experienced in localized ear areas and general comments suggest that there are additional factors at play, e.g., weight, that do not allow dismissal of the efficacy of earplugs.

### Acoustic comfort in four conditions

Increased comfort, decreased discomfort, and increased preference scores for the conditions with ANC headphones (C3) and Earplugs (C4) are an indication that they help make turboprop airplanes (and possibly future electrical propelled airplanes) a more competitive option compared to jet engine airplanes and improve the overall comfort/discomfort experience. The non-significant preference comparison between ANC headphones and jet-engine airplanes further affirms the effectiveness of using such devices as most participants had previous experience with jet-engine airplanes. The fact that passengers are able to choose a sound reduction system such as headphones and earplugs in itself might have a positive effect on comfort and discomfort as Bouwens et al.^26^ state that being in control of noise levels can improve airplane comfort.

Between earplugs and ANC headphones, there is a general preference for the ANC headphones. But it cannot be concluded if this is because of the comfort of the wearable or because of the better blocking of the sound. In Q6 it is seen that the impact of noise on discomfort is the same for both conditions, but in the open questions ‘the effectiveness of noise cancellation’ was mentioned as an advantage of ANC. For comfort, ANC headphones score significantly better than earplugs, but for discomfort and preference, this is not true. It could be that wearing headphones has a larger impact on discomfort than on comfort, which can be explained by discomfort being more associated with pain or numbness, and comfort is more associated with relaxation and emotional well-being.^33^ Although some device-specific discomfort aspects could be clearly defined, future research might include a condition without noise. This way the impact of wearable-specific discomfort factors and the environment factor ‘noise’ on overall comfort and discomfort could be split up and clarified. Additionally, the effect of time on the willingness to use these devices could be further explored. Within the duration of wearing the devices (45 min.), in some areas a significant increase over time was observed. In a longer flight scenario, this effect might lead to higher discomfort levels.

### Factors that influence the choice between ANC headphones and earplugs

The ELD question together with the text analysis supported identifying specific discomfort areas. They also gave insights about participants’ preference towards ANC headphones or earplugs. The headphones create more discomfort around the ear and above the head while the earplugs create more discomfort inside the ear. This was further confirmed by the open questions (e.g., neck pain, pressure on the head and around the ears, or an unfamiliar feeling in the Concha). The preference is personal and individuals can have strong reasons not to wear headphones or earplugs (e.g., feeling of low air pressure with ANC headphones or itchy earplugs). Thus providing a choice to passengers is preferred. These findings correspond with the proposed guidelines from Hsu et al.^34^ to improve the design of hearing protection. Hsu et al.^34^ proposes the following attention points to improve comfort*: Airtightness, weight, heat sinking ability, texture, headband force, and improving possibilities to converse*.

Another factor mentioned as a disadvantage of the ANC headphones was ‘a feeling of low air pressure’. Butterworth & Dragan^35^ describe this phenomenon as “eardrum suck”. In their test, 52% experienced this effect. This effect is psychosomatic, there is no measurable air pressure difference. This is something to consider as well when providing ANC headphones to passengers.

### Research methods

Vink et al.^6^ reported that the factors influencing discomfort in a turboprop airplane (in order of importance) are: noise, vibration, seat, temperature, space, air quality, and lighting. In our research for the condition with only turboprop noise, this order is: Noise, seat, vibration, space, temperature, air quality, and lighting. Although there are slight differences, the top 3 is highly comparable. As the main objective of this research is ‘acoustic comfort’ this outcome shows that the research setup for this purpose is sufficient. The research of Vink et al.^6^ took place in an actual airplane with more airplane-specific vibrations, which could explain the differences in the vibration outcome.

During the analysis, we present both the outcomes with and without a Bonferroni correction. However, in the subsequent discussion and conclusion, we primarily focus on the uncorrected results. While there exists a heightened risk of type one errors, given the research area's alignment with existing literature in the area of comfort research,^36,37^ it might not be absolutely imperative to avoid type one errors.^38^ On the other side, with a potentially increased likelihood of type two errors, there might be a chance of overlooking the opportunities introduced by using earplugs.

The dB(A) was slightly different across the seats. Although in a real flight there are also differences in dB(A) between seat locations,^17^ this fact made it impossible to make a between subject comparison. Therefore, a future study should consider either further equalize SPL levels across seats or search for a relation between seat location and acoustic comfort experience.

In evaluating the performance of the hearing protection a subjective approach was taken, by using a questionnaire. Although appropriate in this context, as the perception of passengers will eventually define their satisfaction, scientifically it could be relevant to add an objective dimension. Valentin et al.^27^ underscored the importance of objective measurements when evaluating the impact of sound environments on performance and effectiveness of hearing protection. Thus it is relevant to quantify the performance of these specific devices in absolute values in this specific use case. A microphone in a real ear technique (MIRE), or a manikin as described by respectively ISO 11904-1 and ISO 11094-2^39,40^ could be used to measure the sound level on the inside of the hearing protection close to the ear.

The body map of the ear seems quite useful in this study. Further research might be needed to study the usefulness of the ear sensitivity. Also, Smulders et al.^41^ states that future work might want to investigate on and around the ears for headphone design.

### Design implications

The use of ANC headphones in daily life has gained significant popularity. However, it is important to recognize that not everyone has access to ANC headphones. Airlines can play a crucial role in enhancing the comfort of passengers and increasing their willingness to fly on turboprop aircraft by providing ANC headphones. It is worth noting that the effectiveness of ANC headphones in specific situations can vary based on the brand and model of the headphones. In fact, using an inappropriate model may even lead to an increase in sound levels (dB(A)).^22^ Therefore, it is advisable for airlines to provide specific tested models of ANC headphones and the associated instructions to passengers to ensure optimal noise cancellation and improve the comfort of passengers.

As a relatively low-cost solution, it might be beneficial for airlines to offer earplugs since a positive effect is also shown for earplugs. However, the best type of earplug and how it should be introduced needs to be studied further as Casali^42^ states that proper fit to the user's ears and training in insertion procedures are critical to the success of earplugs.

When larger adjustments in the airplane cabin are possible, it could be considered to look into placing noise cancellation into the head rest or seat^43^ or integrating noise cancelling panels into the airplane interior,^44^ and in this way reduce noise and device specific discomfort.

### Limitations

The choice to compare ANC headphones and earplugs was made to provide a contrast between an ANC device and a readily implementable noise reduction option. In future research, it may be beneficial to explore a wider range of options, including ANC earbuds and passive noise-canceling headphones.

During the research, the participants did not get a specific task. They were only asked not to do any tasks involving sound, because of the nature of the research. It should be considered that the specific activity of participants can impact the outcomes, for instance, Smulders & Vink^45^ describe that under higher workload, participants reported higher acoustic discomfort. Certain activities such as viewing a VR environment, can also serve as a distraction to other discomfort factors, although more effective for distracting from a restricted space than noise disturbances.^46^ In this study the advantage of using ANC headphones in concentrating tasks was mentioned by participants. Concurrently this study did not consider social factors during the test. Participants were allowed to engage in conversations with each other, but the impact of these interactions on device preference was not recorded, although in the open questions difficulty in communication was mentioned for both ANC headphones and earplugs as a disadvantage. On the work floor having communication difficulties is considered uncomfortable,^34^ but according to others background conversations are found annoying.^26^ Moreover, ANC devices offer extra options for entertainment, which could be a valuable area of investigation in future research.

Additionally, the earplug choice could be studied further as there are many earplugs available, and differences between them regarding fit, the way of placing them^42^ and application in an aircraft might lead to a specific preferred type and instruction, which was not studied in our research. Besides an instruction for using the earplugs, the placement was not checked by the researchers. Incorrect placement could provide negligible attenuation,^42^ which could have a negative impact on the comfort and discomfort experience, but since the participants did receive instructions for checking a correct fit themselves, this might be a minor risk.

It is also important to note that this test did not take place in a flying airplane, and as such, sound perception may differ from a real-life setting. In a real flight vibrations and sound both could amplify each other.^47^ In this research the participants only experienced sound, and there was for instance an absence of engine vibrations, which could have influenced the outcomes. The test setup involved a directional sound source directed at the back of the participants, and some reported variations in sound perception between their ears. In an actual flight, the sound environment might be more omnidirectional. Additionally, the average age of the participants in this study was approximately 27, and it is known that the sensitivity of human hearing decreases with age.^48^ Considering this, the effects of earplugs and ANC headphones might vary for individuals in different age groups than those studied in this experiment.

## Conclusion

Active noise cancellation headphones and earplugs can improve the comfort experience of passengers in turboprop aircraft. The use of these wearables increases overall comfort, reduces discomfort, and is preferred over not wearing any hearing protection on turboprop flights. Between them, ANC headphones are slightly preferred over earplugs. ANC headphones have a better performance regarding filtering noise, but both options bring their own specific limitations. Participants wearing the ANC headphones experienced more discomfort around the ears and on the head compared to using the earplugs where they experienced more discomfort in the area inside the ear. The preference is personal, and individuals can have strong reasons not to wear headphones or earplugs (eardrum suck in case of ANC headphones, or itchiness in case of earplugs), thus providing a choice to passengers is preferred.

Scientifically, this study shows that it is useful to add a body map of the area in and around the ear to study the effects on (dis)comfort in that area.
